# TRAF6 regulates autophagy and apoptosis of melanoma cells through c‐Jun/ATG16L2 signaling pathway

**DOI:** 10.1002/mco2.309

**Published:** 2023-07-20

**Authors:** Yeye Guo, Xu Zhang, Jie Li, Zhe Zhou, Susi Zhu, Waner Liu, Juan Su, Xiang Chen, Cong Peng

**Affiliations:** ^1^ Department of Dermatology Xiangya Hospital Central South University Changsha China; ^2^ National Engineering Research Center of Personalized Diagnostic and Therapeutic Technology Changsha China; ^3^ Furong Laboratory Changsha China; ^4^ Hunan Key Laboratory of Skin Cancer and Psoriasis Hunan Engineering Research Center of Skin Health and Disease Xiangya Hospital Central South University Changsha China; ^5^ National Clinical Research Center for Geriatric Disorders (Xiangya Hospital) Changsha China

**Keywords:** apoptosis, ATG16L2, autophagy, c‐Jun, melanoma, TRAF6

## Abstract

Autophagy and apoptosis are essential processes that participate in cell death and maintain cellular homeostasis. Dysregulation of these biological processes results in the development of diseases, including cancers. Therefore, targeting the interaction between apoptosis and autophagy offers a potential strategy for cancer therapy. Melanoma is the most lethal skin cancer. We previously found that tumor necrosis factor receptor‐associated factor 6 (TRAF6) is overexpressed in melanoma and benefits the malignant phenotype of melanoma cells. Additionally, TRAF6 promotes the activation of cancer‐associated fibroblasts in melanoma. However, the role of TRAF6 in autophagy and apoptosis remains unclear. In this study, we found that knockdown of TRAF6 induced both apoptosis and autophagy in melanoma cells. Transcriptomic data and real‐time PCR analysis demonstrated reduced expression of *autophagy related 16 like* 2 (*ATG16L2*) in TRAF6‐deficient melanoma cells. *ATG16L2* knockdown resulted in increased autophagy and apoptosis. Mechanism studies confirmed that TRAF6 regulated *ATG16L2* expression through c‐Jun. Importantly, targeting TRAF6 with cinchonine, a TRAF6 inhibitor, effectively suppressed the growth of melanoma cells by inducing autophagy and apoptosis through the TRAF6/c‐Jun/ATG16L2 signaling pathway. These findings highlight the pivotal role of TRAF6 in regulating autophagy and apoptosis in melanoma, emphasizing its significance as a novel therapeutic target for melanoma treatment.

## Graphical abstract

TRAF6 promotes the transcriptional activity of c‐Jun, resulting in the upregulation of ATG16L2. This signaling pathway plays a pivotal role in the regulation of apoptosis and autophagy in melanoma cells. Inhibition of TRAF6 using cinchonine effectively suppresses melanoma cell growth by inducing apoptosis and autophagy. Our findings highlight the therapeutic potential of targeting the TRAF6‐c‐Jun/ATG16L2 axis in melanoma treatment.

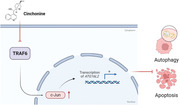

## 1 INTRODUCTION

Cutaneous melanoma is the most lethal skin cancer, accounting for more than three‐quarters of deaths from skin cancers.^1^ During the past two decades, the incidence of melanoma has increased by approximately 2% annually. According to statistics from the American Cancer Society, more than 100,000 new cases of melanoma are expected to be diagnosed and over 7 thousand deaths from melanoma are expected to occur in 2023 (https://www.cancer.org/). Novel treatment strategies for melanoma have been developed, for example, targeted therapies and immunotherapies, which notably prolong the overall survival of melanoma patients.^2^ However, the response rate variability between individuals and drug resistance are commonly observed and result in treatment failure. Therefore, the development of further approaches remains highly important to provide more options for melanoma therapy.

Autophagy is a cellular process involved in the degradation of cellular components within lysosomes under specific conditions. It plays a role in various biological processes related to cancer.^3^ Autophagy has been reported to have a dual role in the development of melanoma. In the early stage, decreased autophagy leads to the loss of protective mechanisms in cells, thereby promoting tumor formation. The restoration of autophagy in advanced melanoma may benefit tumor progression and cause drug resistance,^4^ while increased autophagy could also induce apoptosis in tumor cells under certain conditions.^5^, ^6^ The interplay between autophagy and apoptosis is a complex system. Some specific molecules, such as BCL2 and p62, may be critical links between the two processes. P62 participates in both the selective autophagic degradation and apoptotic pathways by interacting with cell survival‐related proteins such as Caspase‐8, tumor necrosis factor receptor‐associated factor 6 (TRAF6), and extracellular signal‐regulated kinase (ERK).^7^, ^8^, ^9^, ^10^ TRAF6, belonging to the tumor cecrosis factor (TNF) receptor‐associated factor family, is an important signaling molecule that plays a critical role in regulating various cellular processes, including the immune response, inflammation, and cell survival. TRAF6 interacts with several downstream signaling pathways, including the c‐Jun N‐terminal kinase (JNK) and NF‐kB pathways,^11^ which further regulate the transcription of genes involved in cellular processes. For example, TRAF6 can activate the c‐Jun pathway by promoting the phosphorylation and activation of JNK, or interact with c‐Jun directly and promote its stabilization by preventing its degradation.^12^, ^13^ It has been documented that the c‐Jun pathway participates in autophagy by modulating the expression of mammalian target of rapamycin (mTOR), a negative regulator of autophagy, or by activating the transcription factor EB (TFEB), a key regulator of lysosomal biogenesis and autophagy.^14^, ^15^ Our previous work suggests that TRAF6 is highly expressed in melanoma and enhances its invasion and metastasis abilities.^16^ In addition, TRAF6 induces the activation of fibroblasts to cancer‐associated fibroblasts in the tumor microenvironment, therefore promoting the malignant phenotype of melanoma cells.^17^ Nevertheless, the precise involvement of TRAF6 in the autophagic and apoptotic processes within melanoma cells remains uncertain.

Our transcriptomic data suggest that knockdown of TRAF6 regulates the autophagy and apoptosis pathways in melanoma cells. Herein, we found that TRAF6 knockdown promoted both apoptosis and autophagy. The expression level of *Autophagy Related 16 Like 2* (*ATG16L2*) was decreased in TRAF6‐deficient melanoma cells. ATG16L2, a member of the ATG16 protein family, has been documented to participate in the process of selective autophagy, specifically targeting organelles and proteins in intestinal cells.^18^ However, the exact role of ATG16L2 in the autophagy process in the context of cancer is not fully understood. Here, we found that suppressing ATG16L2 expression led to elevated apoptosis and autophagy in melanoma cells. The mechanistic investigation further revealed that TRAF6 regulated the expression of *ATG16L2* through c‐Jun. We next found that cinchonine, a TRAF6 inhibitor, suppressed melanoma tumor growth by enhancing autophagy and leading to apoptosis through the c‐Jun/ATG16L2 pathway. In this research, we present novel findings regarding the function of TRAF6 in apoptosis and autophagy. Specifically, we provide the first evidence demonstrating that TRAF6 is involved in both of these cellular processes. Additionally, we show for the first that ATG16L2 is implicated in autophagy and is regulated by the TRAF6/c‐Jun pathway in melanoma cells. These findings indicate that targeting the TRAF6/c‐Jun/ATG16L2 axis represents a promising therapeutic target for the treatment of melanoma.

## 2 RESULTS

### 2.1 TRAF6 regulates the proliferation of melanoma cells

**FIGURE 1:**
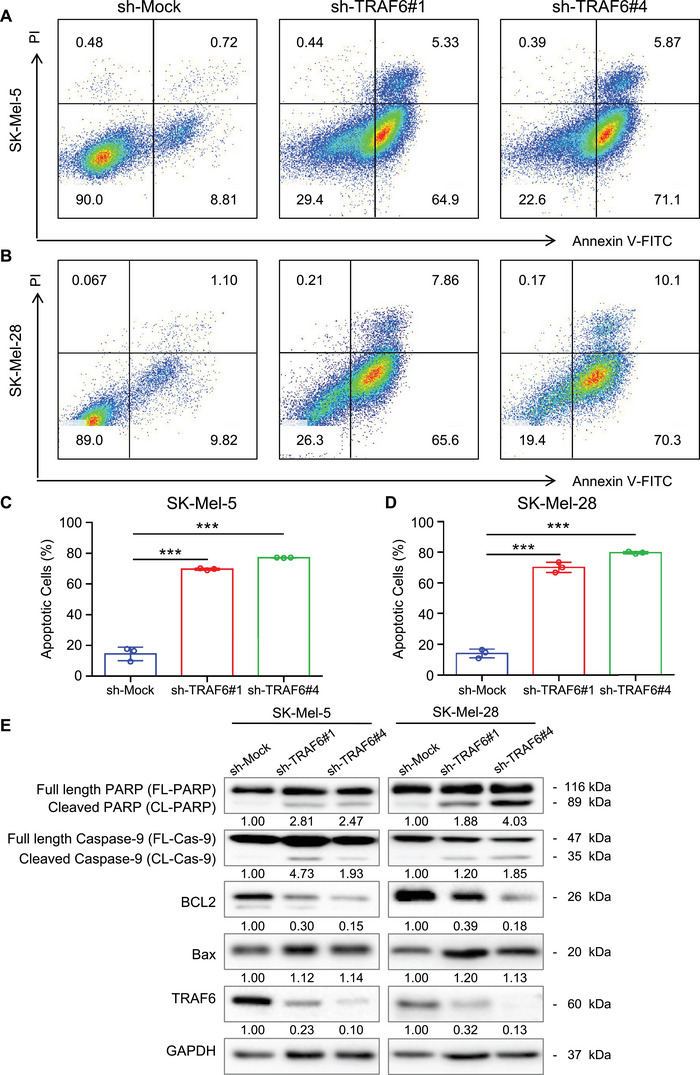
Knockdown with TRAF6 induces apoptosis in melanoma cells. (A and B) Representative flow cytometry dot plot showing the apoptotic cells of control and TRAF6‐knockdown melanoma cells. SK‐Mel‐5 and SK‐Mel‐28 were infected with scramble or sh‐TRAF6 lentivirus for 12 h and incubated with puromycin for 24 h. After another 24 h incubation with complete culture media without puromycin, Annexin V‐FITC and PI staining SK‐Mel‐5 (A) and SK‐Mel‐28 (B) were performed as described in section *Materials and Methods*, and detected by flow cytometry. The bottom left indicates normal cells, the top left indicates necrotic cells, the bottom right shows early apoptotic cells and the top right shows late apoptotic cells (numbers marked in the quadrants represent the respective percentages of total cells). (C and D) Bar charts of apoptotic cell percentage of melanoma cells. Data are presented as the mean ± SD (*n* = 3). Significant differences were evaluated using one‐way ANOVA, ***p < 0.001. (E) Knocking down TRAF6 increases the expression of apoptotic markers. Whole cell lysate of SK‐Mel‐5 (left panel) and SK‐Mel‐28 (right panel) knockdown with TRAF6 were extracted and subjected to immunoblot analysis using antibodies to cleaved PARP, cleaved Caspase‐9, BCL2, Bax and TRAF6 as described in section *Materials and Methods*, GAPDH was used as control.

As we previously demonstrated that TRAF6 was overexpressed in melanoma cells, we expressed short hairpin (sh)‐TRAF6 in the human melanoma cell lines SK‐Mel‐5 and SK‐Mel‐28. Interestingly, we found that the growth of these melanoma cells was significantly inhibited. Therefore, we detected apoptosis in SK‐Mel‐5 and SK‐Mel‐28 cells by Annexin V staining, as illustrated in Figures 1A and B. The apoptosis rate increased fivefold in melanoma cells after knockdown of TRAF6 (apoptosis rate: 60–80%) compared with that in sh‐Mock cells (10–20%) (Figures 1C and D). During cell death, the poly (adenosine diphosphate‐ribose) polymerase‐1 (PARP‐1) enzyme facilitates the repair of damaged deoxyribonucleic acid (DNA) and cleavage of PARP‐1 is a hallmark of apoptosis.^19^, ^20^ In addition to PARP‐1, BCL2 and caspase family members also regulate cell death.^21^ Hence, we measured the expression of these apoptosis‐related proteins by western blotting, which showed that the levels of cleaved PARP, and cleaved Caspase‐9 were significantly increased and the level of BCL2 was greatly decreased in TRAF6‐knockdown melanoma cells (Figure 1E). These results suggested that TRAF6 is involved in the proliferation of melanoma cells.

### 2.2 Knockdown of TRAF6 induces autophagy in melanoma cells

**FIGURE 2:**
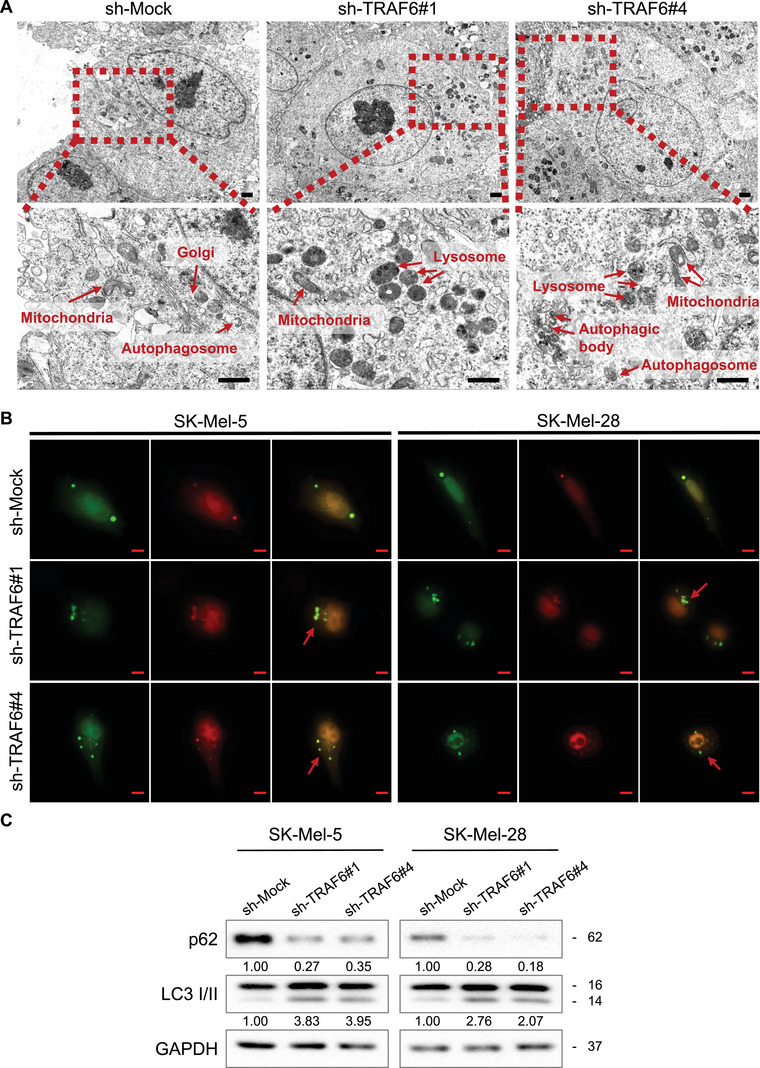
Knocking down of TRAF6 induces autophagy in melanoma cells. (A) Autophagic organelles detected by transmission electron microscopy in melanoma cells. Control cells (left panel) and TRAF6‐knockdown SK‐Mel‐5 melanoma cells (middle and right panel) were prepared and objected to electron microscopy as described in section *Materials and Methods*. The red arrowheads indicate the following organelles: autophagosomes surrounded by a double‐membrane with undigested cytoplasmic material inside; the swollen mitochondria with distorted or disorganized cristae; the enlarged lysosome with electron‐dense material. The scale bar = 1 μm. (B) Knockdown of TRAF6 increases the number of autophagosomes in melanoma cells. The mCherry‐GFP‐LC3B puncta formation assays were performed in TRAF6‐deficient SK‐Mel‐5 (left panel) and SK‐Mel‐28 (right panel) cells as described in section *Materials and Methods*. Representative images showed the distribution of LC3II during the process of autophagy in melanoma cells transfected with mCherry‐GFP‐LC3B plasmid. The red spots signify autophagy lysosomes. The red arrow indicated autophagosomes. The scale bar = 10 μm. (C) Knockdown of TRAF6 induces the expression of autophagic markers. Whole cell lysate of SK‐Mel‐5 (left panel) and SK‐Mel‐28 (right panel) knockdown with TRAF6 were extracted and subjected to immunoblot analysis using antibodies to p62 and LC3 I/II as described in section *Materials and Methods*, GAPDH was used as control.

The interplay between apoptosis and autophagy is widely acknowledged. Increased autophagy can result in induction of apoptosis through lysosomal activity.^22^ Considering this observation, we detected autophagy after knocking down TRAF6. Interestingly, transmission electron microscopy (TEM) analysis suggested that many autophagosomes and autophagic bodies were formed in sh‐TRAF6 melanoma cells, as shown in Figure 2A. LC3 is well recognized as a key protein in autophagic activity^23^; therefore, we evaluated the formation of LC3 puncta and observed an elevation in the number of autophagosomes in cells where TRAF6 was knocked down (Figure 2B). Elevated LC3 II and decreased p62, which are indicators of autophagy,^24^ were found through western blotting (Figure 2C). These results indicated that autophagy was activated when TRAF6 was knocked down in melanoma cells.

### 2.3 The influence of TRAF6 on gene expression profiles in melanoma cells

**FIGURE 3:**
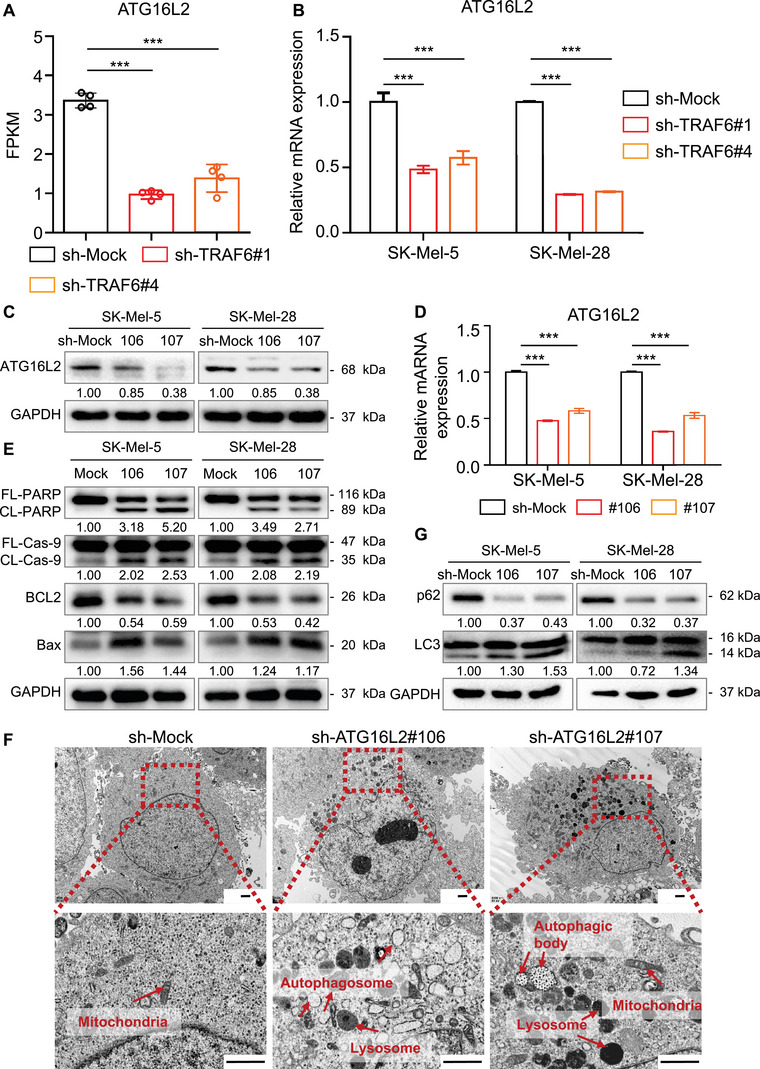
ATG16L2 is reduced in TRAF6‐deficient melanoma cells and regulates autophagy. (A) Fragments per kilobase of exon per million mapped fragments (FPKM) of ATG16L2 is decreased in sh‐TRAF6#1 and sh‐TRAF6#4 melanoma cells. RNA sequencing was performed previously, ***p < 0.001. (B) The expression of ATG16L2 is downregulated in TRAF6‐knockdown melanoma cells. Total RNA was extracted from SK‐Mel‐5 and SK‐Mel‐28 cells, and rt‐PCR was then performed as described in section *Materials and Methods*. The data from multiple experiments are expressed as the mean ± SD (*n* = 3). Significant differences were evaluated using two‐way ANOVA, ***p < 0.001. (C and D) Generation of ATG16L2‐knockdown melanoma cells. SK‐Mel‐5 and SK‐Mel‐28 were infected with sh‐ATG16L2#106 or sh‐ATG16L2#107 lentiviral particles. (C) Whole cell lysate of indicated cells were extracted and subjected to immunoblot analysis using antibodies to ATG16L2 as described in section *Materials and Methods*, GAPDH was used as control. (D) Relative mRNA level of ATG16L2 in melanoma cells. Total RNA was extracted and rt‐PCR was then performed as described in section *Materials and Methods*. The data from multiple experiments are expressed as the mean ± SD (*n* = 3). Significant differences were evaluated using two‐way ANOVA, ***p < 0.001. (E) Knockdown of ATG16L2 induces apoptosis in melanoma cells. Whole cell lysate of sh‐Mock, sh‐ATG16L2#106 and sh‐ATG16L2#107 cells were extracted and subjected to immunoblot analysis using antibodies to Cleaved PARP, Caspase‐9, BCL2, and Bax as described in section *Materials and Methods*, GAPDH was used as control. (F and G) ATG16L2‐deficient melanoma cells exhibited higher autophagy level. (F) Autophagic organelles detected by TEM in melanoma cells. Sh‐Mock cells (F left panel), sh‐ATG16L2#106 (F middle panel), and sh‐ATG16L2#107 (F right panel) cells were prepared and objected to electron microscopy as described in section *Materials and Methods*. The red arrowheads indicate the following organelles: autophagosomes, mitochondria, and lysosomes. The scale bar = 1 μm. (G) Whole cell lysate of sh‐Mock, sh‐ATG16L2#106, and sh‐ATG16L2#107 cells were extracted and subjected to immunoblot analysis using antibodies to p62 and LC3 I/II as described in section *Materials and Methods*, GAPDH was used as control.

To gain deeper insights into the impact of TRAF6 on melanoma cell proliferation, we performed ribonucleic acid (RNA) sequencing of SK‐Mel‐5 cells as described previously.^17^ We performed Kyoto Encyclopedia of Genes and Genomes pathway analysis to analyze the differentially expressed genes identified in our study. The results suggested that the cell growth and death pathway class was altered in TRAF6‐knockdown melanoma cells (Figure S1). Since autophagy was induced, we examined the expression of autophagy‐related genes in the sequencing database. Interestingly, the fragments per kilobase of transcript per million mapped reads (FPKM) values of autophagy‐related genes such as ATG16L2, ATG4C, ATG2B, ATG10, ATG14, ATG3, ATG3, and BECN1^25^, ^26^, ^27^ were significantly altered in SK‐Mel‐5 cells with TRAF6 knockdown (Figures 3A and S2). RT‐PCR analysis further identified the differential expression of these genes (Figure S3), among which ATG16L2 exhibited the greatest decrease in expression in TRAF6‐deficient cells, consistent with the sequencing data (Figure 3B). Analysis of the RNA sequencing data revealed that TRAF6 exerts a regulatory influence on the expression of genes related to autophagy, with ATG16L2 exhibiting the most significant alteration.

### 2.4 Inhibition of ATG16L2 induces autophagy and apoptosis in melanoma cells

ATG16L2 is an autophagy‐related protein, but its role in autophagy has not been clarified. ATG16L1‐deficient macrophages were found to exhibit impaired autophagy and promote the secretion of inflammatory cytokines.^28^ In contrast, knockdown of ATG16L2 was found to increase autophagy in pancreatic acinar cells.^29^ Since we found a significant change in ATG16L2 expression in TRAF6‐knockdown melanoma cells, further investigation of the role of ATG16L2 was deemed highly important. Therefore, we knocked down ATG16L2 in melanoma cell lines, including SK‐Mel‐5 and SK‐Mel‐28 (Figures 3C and D). First, we measured the protein levels of apoptosis markers in ATG16L2‐deficient cells. Interestingly, we observed a significant increase in the expression of proapoptotic proteins including cleaved PARP, cleaved Caspase‐9 and Bax, as well as decreased levels of antiapoptotic proteins such as BCL2 (Figure 3E). Next, we tried to determine the function of ATG16L2 in melanoma cells. The level of autophagy was evaluated by TEM, and numerous phagosomes were observed in ATG16L2‐knockdown melanoma cells (Figure 3F). Consistent with our previous findings, these cells expressed a higher level of LC3 II and a decreased level of p62 (Figure 3G), indicating the regulatory role of ATG16L2 in autophagy. These results support the notion that inhibition of ATG16L2 plays a role in modulating autophagy and apoptosis in melanoma cells.

### 2.5 Cinchonine induces apoptosis and autophagy in melanoma cells

Here, we found that TRAF6 participates in the survival pathway in melanoma cells through ATG16L2, and our previous studies demonstrated that TRAF6 promotes the malignant phenotype of melanoma cells.^16^, ^17^ Considering these findings, targeting TRAF6 could be a promising therapeutic approach for melanoma. Hence, we treated melanoma cells with cinchonine, a compound from the Cinchona alkaloid family that binds to the RING domain of TRAF6 and affects its function.^30^

**FIGURE 4:**
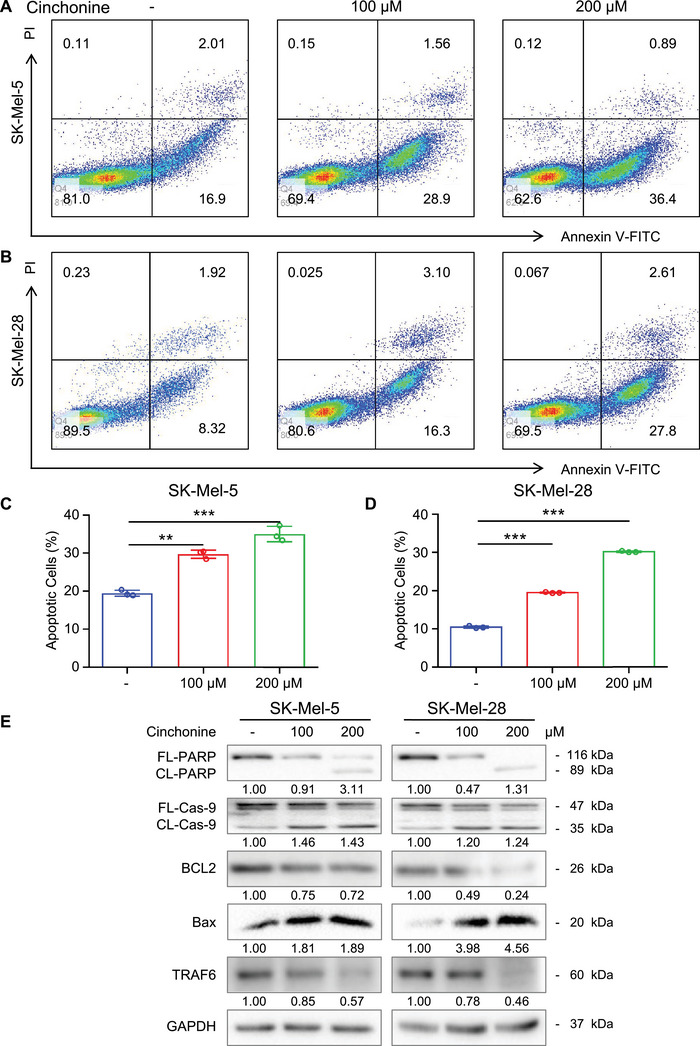
Cinchonine promotes the apoptosis of melanoma cells. (A and B) Representative flow cytometry dot plot showing the apoptotic cells of melanoma cells treated with vehicle or cinchonine. SK‐Mel‐5 and SK‐Mel‐28 were treated with vehicle (left panel), 100 μM (middle panel), or 200 μM (right panel) cinchonine for 24 h. Annexin V‐FITC and PI staining SK‐Mel‐5 (A) and SK‐Mel‐28 (B) were performed as described in section *Materials and Methods*, and detected by flow cytometry. (C and D) Bar charts of apoptotic cell percentage of melanoma cells. Data are presented as the mean ± SD (*n* = 3). Significant differences were evaluated using one‐way ANOVA, **p < 0.01, ***p < 0.001. (E) Cinchonine elevates the expression of apoptotic markers. Whole cell lysate of SK‐Mel‐5 (left panel) and SK‐Mel‐28 (right panel) treated with vehicle, 100 or 200 μM cinchonine were extracted and subjected to immunoblot analysis using antibodies to cleaved PARP, cleaved Caspase‐9, BCL2, Bax, and TRAF6 as described in section *Materials and Methods*, GAPDH was used as control.

**FIGURE 5:**
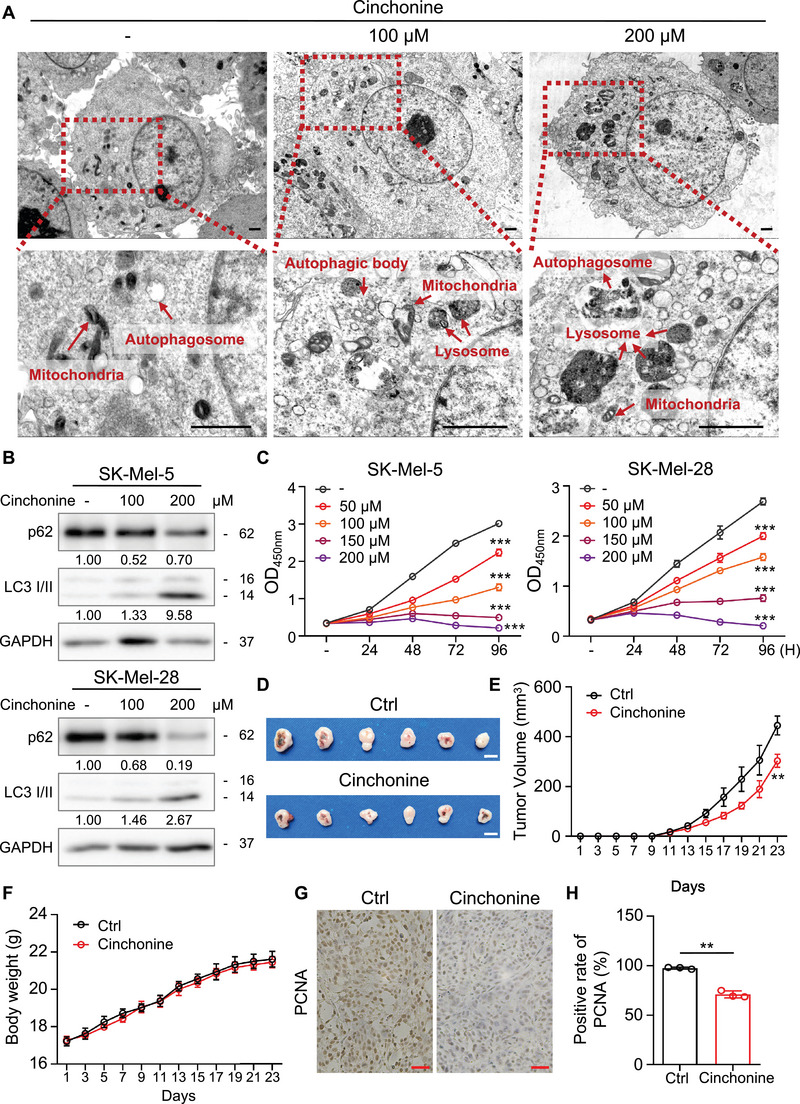
Cinchonine induces autophagy and suppresses tumor growth of melanoma in vitro and in vivo. (A) Autophagic organelles captured by transmission electron microscopy in melanoma cells treated with cinchonine. Control cells (left panel), 100 μM (middle panel), or 200 μM (right panel) cinchonine‐treated SK‐Mel‐5 cells were prepared and objected to electron microscopy as described in section *Materials and Methods*. The red arrowheads indicate the following organelles: autophagosomes, mitochondria, and lysosomes. The scale bar = 1 μm. (B) Treatment with cinchonine increases the expression of autophagic markers. Whole cell lysate of SK‐Mel‐5 (B upper panel) and SK‐Mel‐28 (B lower panel) treated with cinchonine were extracted and subjected to immunoblot analysis using antibodies to p62 and LC3 I/II as described in section *Materials and Methods*, GAPDH was used as control. (C) Cinchonine inhibits the growth of melanoma cells in vitro. SK‐Mel‐5 (C left panel) and SK‐Mel‐28 (C right panel) were treated with cinchonine (vehicle, 50, 100, 150, and 200 μM). Cells were seeded into 96‐well plates, and cell viability was examined by CCK‐8 kit as described in section *Materials and Methods*. Data from multiple experiments are expressed as the means ± SD. Significant differences were evaluated using two‐way ANOVA, ***p < 0.001. (D and E) Cinchonine suppresses melanoma cell growth in vivo. The melanoma cells (1 × 10^6^ cells) were xenografted into the right flank of nude mice. After tumors grow to 5 × 5 mm^3^, all the mice were randomly divided into two groups (*n* = 6 for each group) and treated with PBS control or cinchonine as described in section *Materials and Methods*. Representative xenografted tumors from melanoma mouse models at 23 days after implantation (D). The scale bar = 1 cm. Tumor growth was measured four times a week (E). The results are shown as the mean tumor volume ± SD. Significant differences were evaluated using two‐way ANOVA, **p < 0.01. (F) Body weight curve of mice treated with cinchonine. Body weight of mice was measured at indicated time points. (G and H) Immunohistochemistry staining of PCNA (1:350) in xenografted melanoma mouse mode tissues as described in section *Materials and Methods*. Representative images were taken (G) and bar chart graphs of PCNA positive rate (%) (H). The scale bar = 100 μm. Data are presented as the mean ± SD (*n* = 4). Significant differences were evaluated using one‐way ANOVA, **p < 0.01.

As expected, both SK‐Mel‐5 and SK‐Mel‐28 cells exhibited an increased apoptosis rate when treated with cinchonine (Figures 4A and B). After treatment with 100 μM cinchonine, 29.7 ± 0.76% of SK‐Mel‐5 and 19.5 ± 0.15% of SK‐Mel‐28 cells were apoptotic. When the cinchonine concentration was increased to 200 μM, apoptosis was detected in 35.02 ± 2.27% of SK‐Mel‐5 and 30.2 ± 0.21% of SK‐Mel‐28 cells (Figures 4C and D), indicating that cinchonine induces apoptosis in melanoma cells in a dose‐dependent manner. Consistent with these results, western blotting showed that the levels of cleaved PARP and cleaved Caspase‐9 were elevated, while the level of BCL2 was decreased (Figure 4E), which further demonstrated that cinchonine induces apoptosis in melanoma cells. Next, TEM was performed in melanoma cells treated with cinchonine. Notably, increased accumulation of autophagic bodies and autophagosomes was observed in cinchonine‐treated melanoma cells (Figure 5A). More autophagosomes were found after treatment with 200 μM cinchonine than after treatment with 100 μM cinchonine. Figure 5B illustrates the effects of cinchonine on the levels of LC3 II and p62, two key markers associated with autophagy. Taken together, these data suggested that treatment with cinchonine induced apoptosis and autophagy in melanoma cells.

### 2.6 Cinchonine inhibits the growth of melanoma cells in vitro and in vivo

To investigate the inhibitory role of cinchonine in cell growth, we treated melanoma cells with different concentrations of cinchonine. The growth of SK‐Mel‐5 and SK‐Mel‐28 cells was reduced by approximately 70% after treatment with 50 μM cinchonine and by 50% after treatment with 100 μM cinchonine. When the concentration was increased to 150 μM, the growth was almost completely inhibited (Figure 5C). Then, we treated xenograft‐bearing mice with cinchonine (100 mg/kg) and found that tumor growth was significantly suppressed (Figures 5D and E). To assess the toxicity of cinchonine, we measured the body weight of mice treated with the compound, as TRAF6 is expressed in various cells (Figure 5F). The data indicate that there was no significant decrease in the weight of the mice, suggesting that cinchonine has a tolerable side effect. Immunohistochemistry was then performed on tumor slices to investigate the role of cinchonine in the expression of TRAF6, c‐Jun, and ATG16L2 in tumor tissue. Our results demonstrated that the expression of TRAF6, ATG16L2, and c‐Jun was inhibited in mice treated with cinchonine compared with the control group. These findings are consistent with the in vitro results (Figure S4). Staining of proliferating cell nuclear antigen (PCNA) suggested the presence of fewer proliferating cells in cinchonine‐treated tumors (Figures 5G and H). Moreover, the expression level of Caspase‐9 was elevated in cinchonine‐treated tumors (Figure S4). Taken together, these results indicated that cinchonine inhibited the proliferation and induces apoptosis of melanoma cells.

### 2.7 Cinchonine suppresses the TRAF6/c‐Jun/ATG16L2 pathway

**FIGURE 6:**
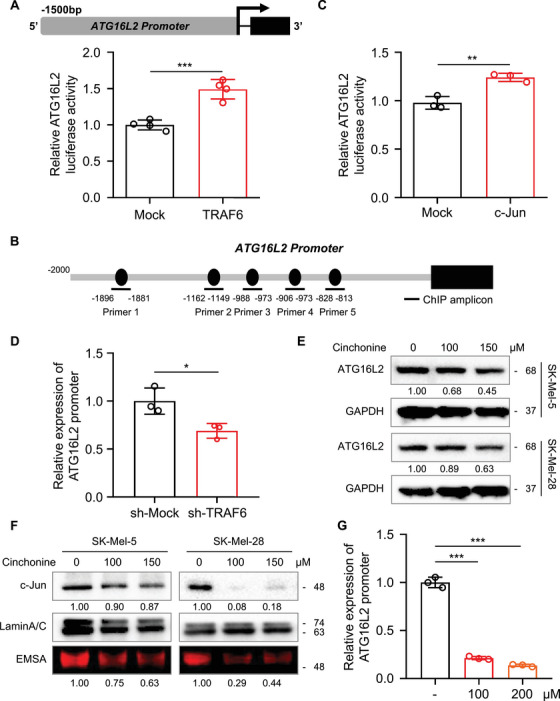
TRAF6 regulates the gene expression profiles of cell growth and death pathway in melanoma cells. (A) Overexpression of TRAF6 increases ATG16L2 luciferase activity. Schematic diagram of pGL3‐ATG16L2‐luc plasmid construction (A upper panel). The *pGL3‐ATG16L2‐luc* and *Renilla luciferase* gene were cotransfected with *Mock or TRAF6 plasmid* into HEK293T cells. After 24 h transfection, *Firefly luciferase* activity was examined through normalized against *Renilla luciferase* activity as described in section *Materials and Methods* (A lower panel). The data from multiple experiments are expressed as the mean ± SD (*n* = 4). Significant differences were evaluated using Student's *t*‐test, ***p < 0.001. (B) Schematic diagram of ATG16L2 promoter and PROMO predicted several binding sites of c‐Jun. (C) ATG16L2 luciferase activity is regulated by c‐Jun in melanoma cells. The *pGL3‐ATG16L2‐luc* and *Renilla luciferase* gene were cotransfected with *Mock or c‐Jun plasmid* into HEK293T cells. 24 h later, *Firefly luciferase* activity and *Renilla luciferase* activity were detected. The data from multiple experiments are expressed as the mean ± SD (*n* = 3). Significant differences were evaluated using Student's *t*‐test, **p < 0.01. (D) Knockdown of TRAF6 attenuates c‐Jun associated with ATG16L2 promoter. ChIP assay was performed to examine the c‐Jun recognition of the ATG16L2 promoter as described in section *Materials and Methods*. The data from multiple experiments are expressed as the mean ± SD (*n* = 3). Significant differences were evaluated using Student's *t*‐test, *p < 0.05. The data of *Primer 1*, *2*, *3*, and *5* were not shown as it did not work. (E) Cinchonine downregulated the expression of ATG16L2 in melanoma cells. Whole cell lysate of SK‐Mel‐5 and SK‐Mel‐28 treated with –, 100, and 150 μM cinchonine were extracted and subjected to immunoblot analysis using antibodies to ATG16L2 as described in section *Materials and Methods*, GAPDH was used as control. (F) Nuclear c‐Jun is decreased after cinchonine treatment in melanoma cells. Nuclear protein was extracted from cinchonine‐treated melanoma cells and subjected to immunoblot analysis using antibodies to c‐Jun or electrophoretic mobility‐shift assays as described in section *Materials and Methods*. Lamin A/C was used as control. (G) Cinchonine reduces c‐Jun associated with ATG16L2 promoter. ChIP assay of cinchonine‐treated melanoma cells with *Primer 4* was performed as described in section *Materials and Methods*. The data from multiple experiments are expressed as the mean ± SD (*n* = 3). Significant differences were evaluated using one‐way ANOVA, ***p < 0.001.

To better understand the regulatory role of TRAF6 in ATG16L2 mRNA expression, we constructed the pGL3‐ATG16L2‐luc plasmid (Figure 6A upper panel). Then pGL3‐ATG16L2‐luc was cotransected with the Mock or TRAF6 plasmid into HEK293T cells, and the transfected cells were subsequently subjected to a luciferase assay. Overexpression of TRAF6 significantly increased the luciferase activity of the ATG16L2 promoter (Figure 6A, lower panel). TRAF6 regulates the p38/c‐Jun pathway in diverse diseases, with effects including modulating inflammatory pathways in keratinocytes,^31^ promoting cell proliferation and progression in colorectal cancer,^32^ and inducing angiogenesis.^33^ According to the prediction from PROMO (http://alggen.lsi.upc.es/), the ATG16L2 promoter contains several potential binding sites for c‐Jun (Figure 6B). Hence, we first performed a luciferase reporter assay and found that the luciferase activity of the ATG16L2 promoter was elevated after overexpression of c‐Jun (Figure 6C). Then, we designed 5 pairs of primers for chromatin immunoprecipitation (ChIP) assays to examine the effect of TRAF6 on the binding of c‐Jun to the ATG16L2 promoter. The results with Primer‐4 indicated that TRAF6 knockdown significantly reduced the occupancy of c‐Jun in the ATG16L2 promoter in SK‐Mel‐28 cells (Figure 6D), suggesting that TRAF6 regulates ATG16L2 expression at the transcriptional level through c‐Jun.

Since cinchonine was reported as a TRAF6 inhibitor and was found to induce apoptosis and autophagy, the inhibitory role of cinchonine in melanoma cells might be associated with the ATG16L2 pathway. Therefore, we first measured the expression of ATG16L2 after treatment with cinchonine. As shown in Figure 6E, treatment with 150 μM of cinchonine notably decreased the level of ATG16L2 in melanoma cells. Next, we evaluated the expression of nuclear c‐Jun after cinchonine treatment. Interestingly, cinchonine significantly suppressed the nuclear accumulation of c‐Jun in both SK‐Mel‐5 and SK‐Mel‐28 cells (Figure 6F). ChIP assays with Primer‐4 further confirmed that cinchonine suppressed c‐Jun binding to the ATG16L2 promote (Figure 6G). Taken together, these results indicated that cinchonine inhibited the growth of melanoma cells through the TRAF6/c‐Jun/ATG16L2 signaling pathway.

## 3 DISCUSSION

Apoptosis and autophagy are different processes of cell death. However, there is no clear boundary between these two processes. It is well documented that apoptosis inhibits the development of cancers by suppressing cell proliferation and metastasis. Apoptosis can be mediated by both the intrinsic pathway after stimulation by stressors such as DNA damage, starvation, and oxidative stress and the extrinsic pathways through the binding of death ligands to death receptors.^34^ Apoptosis of misplaced cancer cells is a key step in inhibiting metastasis. Therefore, several small molecules targeting apoptotic pathways and their components, including the p53 signaling pathway, BCL2 family members, and cIAPs, have been investigated for cancer therapy.^35^, ^36^ Unlike that of apoptosis, the role of autophagy is controversial. Under physiological conditions, autophagy enables the degradation of proteins and organelles to obtain amino acids and macromolecules necessary for cell survival, therefore protecting cells from nutrient deprivation.^37^ During the early stage of cancer, reduced autophagic activity increases the proliferative capacity of cells, hence promoting their malignant transformation. In the advanced stage of cancer, autophagy is restored, benefits the survival of starved cancer cells and leads to drug‐resistance.^38^ Therefore, the role of autophagy is related to the status of cells.

Under some conditions, apoptosis and autophagy are interconnected. For example, the proapoptotic protein TRAIL mediates autophagy during lumen formation.^39^ Ceramide, which triggers apoptosis, induces macroautophagy.^40^ There are several nodes of crosstalk between apoptosis and autophagy such as beclin‐1‐BCL2^41^ and p53. Increased beclin‐1 expression induces BCL2 to trigger apoptosis and enhances the effect of anticancer drugs in cervical cancer.^42^ Suppression of BCL2 can upregulate the expression of beclin‐1, thus leading to apoptosis in breast cancer cells.^43^ P53, a tumor suppressor, is well recognized to induce apoptosis. It plays a complex role in autophagy: cytoplasmic p53 inhibits autophagy, while nuclear p53 activates autophagy.^44^

In this study, we uncovered that TRAF6 exerts regulatory control over both apoptosis and autophagy in melanoma cells. Downregulation of TRAF6 notably increased the apoptosis rate of cancer cells. Autophagic activity was elevated in TRAF6‐deficient melanoma cells, which contained more autophagosomes. TRAF6 is highly expressed in various cancers. TRAF6 suppresses the mitochondrial translocation of p53, thus inhibiting apoptosis in cancer cells.^45^ In osteosarcoma cells, knockdown of TRAF6 was found to increase the apoptosis rate.^46^ In addition to its established role in apoptosis, TRAF6 has been implicated in the regulation of autophagy under specific conditions. Perturbation of the interaction between AMBRA1 and TRAF6 disrupts autophagy by modulating the K63‐linked ubiquitylation of specific proteins.^47^ TRAF6 can also interact with ATG9 thereby inducing autophagy.^48^ Although several studies have investigated the role of TRAF6 in different cancers, its regulatory role in the crosstalk between apoptosis and autophagy remains unknown. In addition to its direct regulation of apoptosis‐ or autophagy‐related genes, TRAF6 also plays a crucial role in promoting cell survival by activating downstream signaling pathways. For example, in human lung cancer cells, TRAF6 interacts with and ubiquitinates PI3K, leading to phosphorylation of AKT at T308 and S473, which endows cancer cells with resistance to apoptosis.^49^ Furthermore, TRAF6 stabilizes myeloid cell leukimia (MCL)‐1 through K63‐linked polyubiquitination, which promotes cell growth.^50^ Notably, TRAF6‐dependent phosphorylation of ERK1/2 induces upregulation of MCL‐1, ultimately suppressing apoptosis in liver cancer cells.^51^ These studies provide compelling evidence for the pivotal role of TRAF6 in regulating cell growth and death. In this study, we present evidence supporting the role of TRAF6 in the downregulation of ATG16L2 through c‐Jun, which ultimately triggers both apoptosis and autophagy in melanoma cells. Moreover, we demonstrate that inhibition of TRAF6 with cinchonine leads to a significant reduction in the growth of melanoma cells both in vitro and in vivo.

Our study has certain limitations. TRAF6 has been previously reported to regulate apoptosis by modulating AP‐1 expression. In this study, we demonstrated that TRAF6 modulated the expression of c‐Jun/ATG16L2, and that knocking down either TRAF6 or ATG16L2 induced apoptosis in melanoma cells. However, the contribution of the ATG16L2 pathway to TRAF6‐induced apoptosis remains unclear. In addition, the specific mechanisms by which ATG16L2 regulates apoptosis require further elucidation.

## 4 CONCLUSION

Taken together, our results indicate that the TRAF6/c‐Jun/ATG16L2 signaling axis plays an essential role in the crosstalk between apoptosis and autophagy in melanoma. Cinchonine, an inhibitor of TRAF6, suppressed the growth of melanoma cells and exhibited antitumor effects, suggesting that targeting TRAF6 is a promising therapeutic strategy in melanoma.

## 5 MATERIALS AND METHODS

### 5.1 Cell lines and cell culture

In this study, we utilized SK‐Mel‐5 and SK‐Mel‐28 human melanoma cells (ATCC HTB‐70; HTB‐72) and HEK293T human kidney cells (ATCC CRL‐3216), following the protocols described in a previous study.^17^ The cells were cultured in Dulbecco's modified Eagle's medium (DMEM; 01‐052‐1A; VivaCell, Shanghai, China), supplemented with 10% fetal bovine serum (04‐001‐1A; VivaCell) and 1% penicillin‐streptomycin (03‐031‐1B; Biological Industries, HaEmek, Israel), and were maintained at a temperature of 37°C with a 5% CO_2_ atmosphere.

### 5.2 Antibodies and reagents

The primary antibodies used were as follows: anti‐TRAF6 [8028Sl; Cell Signaling Technology (CST), MA, USA], anti‐ATG16L2 (1:500, D153556; Sangon Biotech, Shanghai, China), anti‐cleaved PARP (9542S; CST), anti‐cleaved Caspase‐9 (9508S; CST), anti‐BCL2 (12789‐1‐AP; Proteintech, IL, USA), anti‐Bax (5023S; CST), anti‐p62(D199564; Sangon Biotech), anti‐LC3 A/B (4108S; CST), anti‐c‐Jun (9165S; CST), anti‐Lamin A/C (sc‐376248; Santa Cruz, CA, USA), and anti‐GAPDH (60004‐1‐Ig; Proteintech).

### 5.3 Protein preparation and immunoblotting

Cells were lysed using RIPA lysis buffer (P0013D; Beyotime, Shanghai, China), and the protein concentrations were determined using a BCA Protein Assay Kit (Santa Cruz). Nuclear proteins were extracted by NE‐PER Nuclear and Cytoplasmic Extraction Reagents (78835; Thermo Scientific, MA, USA) according to the manufacturer's instructions. Proteins were separated by SDS‐PAGE and electroblotted onto polyvinylidene fluoride membranes (Millipore, Billerica, MA). The membranes were then incubated with specific antibodies, and the immunoreactions were detected using a Bio‐Rad imaging system (Bio‐Rad, USA).

### 5.4 Electrophoretic mobility‐shift assays

A total of 3 μg of nuclear protein was utilized for analysis with an AP‐1 kit (AP‐1 IRDye 700, 829−07925; LI‐COR Biosciences, NE, USA) in accordance with the manufacturer's instructions. Immunoreactions were then detected using the Bio‐Rad imaging system (Bio‐Rad, USA).

### 5.5 Cell counting kit‐8 assay

3000 cells/well were plated in 96‐well plates and incubated with indicated time. After incubation, cell counting kit‐8 (CCK‐8) solution (B34304; Bimake, TX, USA) was added and incubated following the manufacturer's instructions. The absorbance of samples was measured at 450 nm using a spectrophotometer (Beckman, USA). Six replicates of each sample were analyzed.

### 5.6 Annexin V/7‐AAD apoptosis assay

Cells were labeled using the Annexin V‐PE/7‐AAD apoptosis detection kit (CA1030; Solarbio, Beijing, China). In brief, detached cells were suspended and incubated with 5 μL of Annexin V‐PE and 10 μL of 7‐AAD for 15−20 min at room temperature in the dark, then 300 μL of 1× binding buffer was added to stop the reaction. Then the samples were analyzed using flow cytometry within 20 min.

### 5.7 Transmission electron microscopy

Cells were harvested using trypsin and subsequently fixed with 2.5% glutaraldehyde (pH 7.4) at 4°C for 2 h. The samples were then forwarded to Wellbio (Changsha, China) for additional processing, which included embedding, sectioning, staining, and imaging using a JEM1400 transmission electron microscope (JEOL USA, MA, USA).

### 5.8 Lentiviral infection

TRAF6‐deficient and ATG16L2‐deficient cells were generated as described previously.^17^ In brief, sh‐Mock, sh‐TRAF6, or sh‐ATG16L2 plasmids were cotransfected with packaging and envelope plasmids (pSPAX2 and PMD2G) into HEK293T cells. The lentiviral particles were harvested at 48 and 72 h posttransfection and stored at −80°C. Target cells were subsequently infected with the lentiviral particles overnight in the presence of 10 μg/mL polybrene. Replacing the medium with fresh medium containing 2 μg/mL puromycin on the next day. Further experiments were performed using these cells until all control cells became nonviable, which usually occurred within 36−48 h in the presence of puromycin.

### 5.9 Immunohistochemistry

The preparation of paraffin‐embedded tumor slices was performed as described previously.^17^ The primary antibody against PCNA (YM6090; ImmunoWay Biotechnology Company, TX, USA) was used in this study. The average rate of positive PCNA staining was obtained by randomly selecting and analyzing five regions of each sample

### 5.10 Immunofluorescence

Control and TRAF6‐deficient melanoma cells were transfected with mCherry‐GFP‐LC3 for visualization of free autophagosomes (both GFP and mCherry fluorescence) and autophagosomes that had fused with the lysosomes (autolyosomes; mCherry fluorescence only). Zeiss LSM 710 was used to capture fluorescence images (Oberkochen, Germany).

### 5.11 Quantitative reverse transcription‐PCR analysis

The extraction of total RNA and reverse transcription for RT‐PCR were performed as described previously.^17^ The PCR primers used in the study are listed in Table S1. Relative mRNA expression was calculated using the 2^−△△CT^ value.

### 5.12 Luciferase reporter gene assays

The following vectors were used: the pGL3 luciferase reporter vector (E1751; Promega, Madison, WI, USA); the pENTER vector (P100001; Vigene, USA); and the pRLTK Renilla luciferase control reporter vectors (P100001; Promega). HEK293T cells were transfected with pENTER (Mock), the TRAF6 or c‐Jun, and pGL3‐ATG16L2‐luc (constructed in our laboratory), along with pRLTK. TRAF6‐deficient melanoma cells were transfected with the pGL3‐ATG16L2‐luc and pRLTK plasmids. The next day, firefly and Renilla luciferase activity in the cell lysates was measured using the dual luciferase assay kit (E1910; Promega) following the manufacturer's protocol. The luciferase activity in four replicates per transfection was averaged.

### 5.13 ChIP assay

For the ChIP assay, melanoma cells were transduced with the sh‐Mock and sh‐TRAF6 lentiviral vectors or treated with varying doses of cinchonine and subjected to the EZ ChIP KIT protocol (17−371RF; Millipore). Soluble lysates were incubated with 5 μL of an anti‐c‐Jun antibody (9165S; CST) overnight at 4°C in the presence of protease inhibitors. The ATG16L2 promoter regions were amplified by PCR using the primer pairs described in Table S1.

### 5.14 Xenograft model

Six‐week‐old female athymic BALB/c mice were procured from the Department of Laboratory Animals, Central South University. SK‐Mel‐5 cells were harvested and rinsed with PBS buffer, and then suspended in cold serum‐free DMEM at a concentration of 10^7^/mL. 100 μL of cells were subcutaneously injected into the right flanks of the nude mice. Tumors were measured three times per week using calipers, and tumor volumes were calculated using the following formula: length × width × height × 0.52. Tumor tissues were excised, fixed with 10% buffered formalin, and embedded in paraffin for further hematoxylin and eosin staining or immunohistochemical analysis.

### 5.15 Statistical analysis methods

The statistical results are expressed as means ± standard deviations (SD) values, and the significance of differences was determined using Student's *t*‐test, one‐way ANOVA, or two‐way ANOVA. Statistical significance was assumed at *p* < 0.05.

## AUTHOR CONTRIBUTIONS

*Conceptualization*: Xiang Chen and Cong Peng; *data curation*: Yeye Guo, Zhe Zhou, and Susi Zhu; *formal analysis*: Yeye Guo, Xu Zhang, and Waner Liu; *funding acquisition*: Xiang Chen, Cong Peng, Jie Li, Xu Zhang, and Yeye Guo; *investigation*: Yeye Guo, Xu Zhang, and Juan Su; *methodology*: Zhe Zhou and Waner Liu; *project administration*: Juan Su and Cong Peng; *resources*: Juan Su, Xiang Chen, and Cong Peng; *software*: Zhe Zhou and Waner Liu; *supervision*: Xiang Chen and Cong Peng; *validation*: Xu Zhang and Susi Zhu; *visualization*: Yeye Guo and Xu Zhang; *writing—original draft preparation*: Yeye Guo and Xu Zhang; *writing—review and editing*: Jie Li and Cong Peng. All authors have read and approved the final manuscript.

## CONFLICT OF INTEREST STATEMENT

The authors declare that they have no competing interests

## ETHICS STATEMENT

The animal protocol was approved by the Ethics Committee of Xiangya Hospital, Central South University (Changsha, China); approval number: 2022100992.

## Supporting information

Supporting Information

## Data Availability

Datasets related to this article can be found at [https://dataview.ncbi.nlm.nih.gov/object/PRJNA602707?reviewer = 4q16ck35vvs2j3tio8h2bk62s4], hosted at [NCBI].

## References

[1] Gershenwald JE . Stemming the rising incidence of melanoma: calling prevention to action. J Natl Cancer Inst. 2016;108(1). 10.1093/jnci/djv381 PMC6048594 26563358

[2] Jenkins RW , Fisher DE . Treatment of advanced melanoma in 2020 and beyond. J Invest Dermatol. 2021;141(1):23‐31. 32268150 10.1016/j.jid.2020.03.943 PMC7541692

[3] Ke PY . Horning cell self‐digestion: autophagy wins the 2016 nobel prize in physiology or medicine. Biomed J. 2017;40(1):5‐8. 28411883 10.1016/j.bj.2017.03.003 PMC6138592

[4] Guo Y , Zhang X , Wu T , Hu X , Su J , Chen X . Autophagy in skin diseases. Dermatology. 2019;235(5):380‐389. 31269494 10.1159/000500470

[5] Liu Y , Kang X , Niu G , et al. Shikonin induces apoptosis and prosurvival autophagy in human melanoma A375 cells via ROS‐mediated ER stress and p38 pathways. Artif Cells Nanomed Biotechnol. 2019;47(1):626‐635. 30873870 10.1080/21691401.2019.1575229

[6] Sun Z , Zheng L , Liu X , Xing W , Liu X . Sinomenine inhibits the growth of melanoma by enhancement of autophagy via PI3K/AKT/mTOR inhibition. Drug Des Devel Ther. 2018;12:2413‐2421. 10.2147/DDDT.S155798 PMC6084074 30122899

[7] Moscat J , Diaz‐Meco MT . p62 at the crossroads of autophagy, apoptosis, and cancer. Cell. 2009;137(6):1001‐1004. 19524504 10.1016/j.cell.2009.05.023 PMC3971861

[8] Dikic I , Johansen T , Kirkin V . Selective autophagy in cancer development and therapy. Cancer Res. 2010;70(9):3431‐3434. 20424122 10.1158/0008-5472.CAN-09-4027

[9] Kirkin V , McEwan DG , Novak I , Dikic I . A role for ubiquitin in selective autophagy. Mol Cell. 2009;34(3):259‐269. 19450525 10.1016/j.molcel.2009.04.026

[10] Mathew R , Karp CM , Beaudoin B , et al. Autophagy suppresses tumorigenesis through elimination of p62. Cell. 2009;137(6):1062‐1075. 19524509 10.1016/j.cell.2009.03.048 PMC2802318

[11] Li J , Liu N , Tang L , et al. The relationship between TRAF6 and tumors. Cancer Cell Int. 2020;20:429. 32905356 10.1186/s12935-020-01517-z PMC7469280

[12] Yamashita M , Fatyol K , Jin C , Wang X , Liu Z , Zhang YE . TRAF6 mediates Smad‐independent activation of JNK and p38 by TGF‐beta. Mol Cell. 2008;31(6):918‐924. 18922473 10.1016/j.molcel.2008.09.002 PMC2621323

[13] Busch J , Moreno R , de la Vega L , et al. TRAF6 phosphorylation prevents its autophagic degradation and re‐shapes LPS‐triggered signaling networks. Cancers (Basel). 2021;13(14):3618. 34298830 10.3390/cancers13143618 PMC8303406

[14] Sun Y , Chen K , Lin G , Wan F , Chen L , Zhu X . Silencing c‐Jun inhibits autophagy and abrogates radioresistance in nasopharyngeal carcinoma by activating the PI3K/AKT/mTOR pathway. Ann Transl Med. 2021;9(13):1085. 34422997 10.21037/atm-21-2563 PMC8339856

[15] Xu J , Zhang XQ , Zhang Z . Transcription factor EB agonists from natural products for treating human diseases with impaired autophagy‐lysosome pathway. Chin Med. 2020;15(1):123. 33292395 10.1186/s13020-020-00402-1 PMC7684757

[16] Luo Z , Zhang X , Zeng W , et al. TRAF6 regulates melanoma invasion and metastasis through ubiquitination of Basigin. Oncotarget. 2016;7(6):7179‐7192. 26769849 10.18632/oncotarget.6886 PMC4872777

[17] Guo Y , Zhang X , Zeng W , et al. TRAF6 activates fibroblasts to cancer‐associated fibroblasts through FGF19 in tumor microenvironment to benefit the malignant phenotype of melanoma cells. J Invest Dermatol. 2020;140(11):2268‐2279. e11. 32275977 10.1016/j.jid.2020.03.950

[18] Khor B , Conway KL , Omar AS , et al. Distinct tissue‐specific roles for the disease‐associated autophagy genes ATG16L2 and ATG16L1. J Immunol. 2019;203(7):1820‐1829. 31451676 10.4049/jimmunol.1800419 PMC6761021

[19] Dantzer F , Schreiber V , Niedergang C , et al. Involvement of poly(ADP‐ribose) polymerase in base excision repair. Biochimie. 1999;81(1‐2):69‐75. 10214912 10.1016/s0300-9084(99)80040-6

[20] Kaufmann SH , Desnoyers S , Ottaviano Y , Davidson NE , Poirier GG . Specific proteolytic cleavage of poly(ADP‐ribose) polymerase: an early marker of chemotherapy‐induced apoptosis. Cancer Res. 1993;53(17):3976‐3985. 8358726

[21] Brentnall M , Rodriguez‐Menocal L , De Guevara RL , Cepero E , Boise LH . Caspase‐9, caspase‐3 and caspase‐7 have distinct roles during intrinsic apoptosis. BMC Cell Biol. 2013;14:32. 23834359 10.1186/1471-2121-14-32 PMC3710246

[22] Ferri KF , Kroemer G . Organelle‐specific initiation of cell death pathways. Nat Cell Biol. 2001;3(11):E255‐E263. 11715037 10.1038/ncb1101-e255

[23] Tanida I , Ueno T , Kominami E . LC3 and autophagy. Methods Mol Biol. 2008;445:77‐88. 18425443 10.1007/978-1-59745-157-4_4

[24] Bjorkoy G , Lamark T , Pankiv S , Overvatn A , Brech A , Johansen T . Monitoring autophagic degradation of p62/SQSTM1. Methods Enzymol. 2009;452:181‐197. 19200883 10.1016/S0076-6879(08)03612-4

[25] Metlagel Z , Otomo C , Takaesu G , Otomo T . Structural basis of ATG3 recognition by the autophagic ubiquitin‐like protein ATG12. Proc Natl Acad Sci USA. 2013;110(47):18844‐18849. 24191030 10.1073/pnas.1314755110 PMC3839761

[26] Hosokawa N , Sasaki T , Iemura S , Natsume T , Hara T , Mizushima N . Atg101, a novel mammalian autophagy protein interacting with Atg13. Autophagy. 2009;5(7):973‐979. 19597335 10.4161/auto.5.7.9296

[27] Kang R , Zeh HJ , Lotze MT , Tang D . The Beclin 1 network regulates autophagy and apoptosis. Cell Death Differ. 2011;18(4):571‐580. 21311563 10.1038/cdd.2010.191 PMC3131912

[28] Saitoh T , Fujita N , Jang MH , et al. Loss of the autophagy protein Atg16L1 enhances endotoxin‐induced IL‐1beta production. Nature. 2008;456(7219):264‐268. 18849965 10.1038/nature07383

[29] Li N , Wu X , Holzer RG , et al. Loss of acinar cell IKKalpha triggers spontaneous pancreatitis in mice. J Clin Invest. 2013;123(5):2231‐2243. 23563314 10.1172/JCI64498 PMC3635720

[30] Qi Y , Pradipta AR , Li M , et al. Cinchonine induces apoptosis of HeLa and A549 cells through targeting TRAF6. J Exp Clin Cancer Res. 2017;36(1):35. 28231796 10.1186/s13046-017-0502-8 PMC5324264

[31] Jiang R , Xu J , Zhang Y , et al. Ligustrazine alleviates psoriasis‐like inflammation through inhibiting TRAF6/c‐JUN/NFkappaB signaling pathway in keratinocyte. Biomed Pharmacother. 2022;150:113010. 35468584 10.1016/j.biopha.2022.113010

[32] Zhu G , Cheng Z , Huang Y , et al. TRAF6 promotes the progression and growth of colorectal cancer through nuclear shuttle regulation NF‐kB/c‐jun signaling pathway. Life Sci. 2019;235:116831. 31487530 10.1016/j.lfs.2019.116831

[33] Pollet I , Opina CJ , Zimmerman C , Leong KG , Wong F , Karsan A . Bacterial lipopolysaccharide directly induces angiogenesis through TRAF6‐mediated activation of NF‐kappaB and c‐Jun N‐terminal kinase. Blood. 2003;102(5):1740‐1742. 12714497 10.1182/blood-2003-01-0288

[34] Verbrugge I , Johnstone RW , Smyth MJ . SnapShot: extrinsic apoptosis pathways. Cell. 2010;143(7):1192. 1192 e1‐2. 21183080 10.1016/j.cell.2010.12.004

[35] Wong RS . Apoptosis in cancer: from pathogenesis to treatment. J Exp Clin Cancer Res. 2011;30(1):87. 21943236 10.1186/1756-9966-30-87 PMC3197541

[36] Bai L , Wang S . Targeting apoptosis pathways for new cancer therapeutics. Annu Rev Med. 2014;65:139‐155. 24188661 10.1146/annurev-med-010713-141310

[37] Hippert MM , O'Toole PS , Thorburn A . Autophagy in cancer: good, bad, or both? Cancer Res. 2006;66(19):9349‐9351. 17018585 10.1158/0008-5472.CAN-06-1597

[38] Gozuacik D , Kimchi A . Autophagy as a cell death and tumor suppressor mechanism. Oncogene. 2004;23(16):2891‐2906. 15077152 10.1038/sj.onc.1207521

[39] Mills KR , Reginato M , Debnath J , Queenan B , Brugge JS . Tumor necrosis factor‐related apoptosis‐inducing ligand (TRAIL) is required for induction of autophagy during lumen formation in vitro. Proc Natl Acad Sci USA. 2004;101(10):3438‐3443. 14993595 10.1073/pnas.0400443101 PMC373480

[40] Scarlatti F , Bauvy C , Ventruti A , et al. Ceramide‐mediated macroautophagy involves inhibition of protein kinase B and up‐regulation of beclin 1. J Biol Chem. 2004;279(18):18384‐18391. 14970205 10.1074/jbc.M313561200

[41] Liang XH , Kleeman LK , Jiang HH , et al. Protection against fatal Sindbis virus encephalitis by beclin, a novel Bcl‐2‐interacting protein. J Virol. 1998;72(11):8586‐8596. 9765397 10.1128/jvi.72.11.8586-8596.1998 PMC110269

[42] Sun Y , Liu JH , Jin L , et al. Over‐expression of the Beclin1 gene upregulates chemosensitivity to anti‐cancer drugs by enhancing therapy‐induced apoptosis in cervix squamous carcinoma CaSki cells. Cancer Lett. 2010;294(2):204‐210. 20207475 10.1016/j.canlet.2010.02.001

[43] Akar U , Chaves‐Reyez A , Barria M , et al. Silencing of Bcl‐2 expression by small interfering RNA induces autophagic cell death in MCF‐7 breast cancer cells. Autophagy. 2008;4(5):669‐679. 18424910 10.4161/auto.6083

[44] Crighton D , Wilkinson S , O'Prey J , et al. DRAM, a p53‐induced modulator of autophagy, is critical for apoptosis. Cell. 2006;126(1):121‐134. 16839881 10.1016/j.cell.2006.05.034

[45] Zhang X , Li CF , Zhang L , et al. TRAF6 restricts p53 mitochondrial translocation, apoptosis, and tumor suppression. Mol Cell. 2016;64(4):803‐814. 27818144 10.1016/j.molcel.2016.10.002 PMC5541903

[46] Meng Q , Zheng M , Liu H , et al. TRAF6 regulates proliferation, apoptosis, and invasion of osteosarcoma cell. Mol Cell Biochem. 2012;371(1‐2):177‐186. 22886393 10.1007/s11010-012-1434-4

[47] Nazio F , Strappazzon F , Antonioli M , et al. mTOR inhibits autophagy by controlling ULK1 ubiquitylation, self‐association and function through AMBRA1 and TRAF6. Nat Cell Biol. 2013;15(4):406‐416. 23524951 10.1038/ncb2708

[48] Tang HW , Liao HM , Peng WH , Lin HR , Chen CH , Chen GC . Atg9 interacts with dTRAF2/TRAF6 to regulate oxidative stress‐induced JNK activation and autophagy induction. Dev Cell. 2013;27(5):489‐503. 24268699 10.1016/j.devcel.2013.10.017

[49] Wang Z , Liu Y , Huang S , Fang M . TRAF6 interacts with and ubiquitinates PIK3CA to enhance PI3K activation. FEBS Lett. 2018;592(11):1882‐1892. 29729098 10.1002/1873-3468.13080

[50] Choi YB , Harhaj EW . HTLV‐1 tax stabilizes MCL‐1 via TRAF6‐dependent K63‐linked polyubiquitination to promote cell survival and transformation. PLoS Pathog. 2014;10(10):e1004458. 25340740 10.1371/journal.ppat.1004458 PMC4207805

[51] Song P , Yang S , Hua H , et al. The regulatory protein GADD34 inhibits TRAIL‐induced apoptosis via TRAF6/ERK‐dependent stabilization of myeloid cell leukemia 1 in liver cancer cells. J Biol Chem. 2019;294(15):5945‐5955. 30782845 10.1074/jbc.RA118.006029 PMC6463698

