# Supplementary material for: TRAF6 regulates autophagy and apoptosis of melanoma cells through c‐Jun/ATG16L2 signaling pathway
Source: MedComm (2020). 2023 Jul 20;4(4):e309. doi: 10.1002/mco2.309 (PMC10357248; doi:10.1002/mco2.309)
Supplement: Supplementary file 1 — Supporting Information [file MCO2-4-e309-s001.pdf]

Title: TRAF6 regulates autophagy and apoptosis of melanoma cells through c-Jun/ATG16L2 signaling pathway

Running title: TRAF6 regulates autophagy and apoptosis

Authors: Yeye Guo[1-5#], Xu Zhang[1-5#], Jie Li[1-5], Zhe Zhou[1-5], Susi Zhu[1-5], Waner Liu[1-5], Juan Su[1-5], Xiang Chen[1-5*], Cong Peng[1-5*]

[#] These authors contributed equally to this work.

[*] These authors contributed equally to this work.

Affiliations:

[1] Department of Dermatology, Xiangya Hospital, Central South University, Changsha, China, 410008

[2] National Engineering Research Center of Personalized Diagnostic and Therapeutic Technology

[3] Furong Labratory，Changsha, Hunan, China

[4] Hunan Key Laboratory of Skin Cancer and Psoriasis and Hunan Engineering Research Center of Skin Health and Disease, Xiangya Hospital, Central South University, Changsha, China, 410008

[5] National Clinical Research Center for Geriatric Disorders (Xiangya Hospital), Changsha, China, 410008

Supplementary Figure Legends

Figure S1. TRAF6 regulates the gene expression profiles of cell growth and death pathway in melanoma cells.

(**A**) Kyoto Encyclopedia of Genes and Genomes (KEGG) pathway classification of differential expression genes in TRAF6-deficient cells. The x-axis is the number of genes, and the y-axis is the enriched pathways. RNA sequencing data of TRAF6-knockdown melanoma cells were acquired from NCBI (Accession: PRJNA602707) as described in *Materials and Methods*.

Figure S2. The expression profile of autophage-related genes after knockdown of TRAF6.

Expression profile of autophagy-related genes in sh-Mock, sh-TRAF6#1 and sh-TRAF6#4 cells. FPKM of *ATG4C, ATG2B, ATG3, ATG10, ATG14, ATG101* and *BECN1* in TRAF6-deficient melanoma cells. The data from multiple experiments are expressed as the mean ± SD ($n = 4$). Significant differences were evaluated using one-way ANOVA, *$p < 0.05$, **$p < 0.01$, ***$p < 0.001$.

Figure S3. The relative expression of autophage-related genes after knockdown of TRAF6.

Relative mRNA level of *ATG4C, ATG2B, ATG3, ATG10, ATG14, ATG101* and *BECN1* genes in melanoma cells. Total RNA was extracted and rt-PCR was then performed as described in *Materials and Methods*. The data from multiple experiments are expressed as the mean ± SD ($n = 3$). Significant differences were evaluated using two-way ANOVA, *$p < 0.05$, **$p < 0.01$, ***$p < 0.001$.

Figure S4. TRAF6, ATG16L2, and c-Jun was inhibited in mice treated with cinchonine.

Immunohistochemistry staining of TRAF6, ATG16L2, c-Jun and Caspase-9 in xenografted

melanoma mouse mode tissues as described in Materials and Methods. Representative images were taken. The scale bar = 100 μm.

Figure S1

A

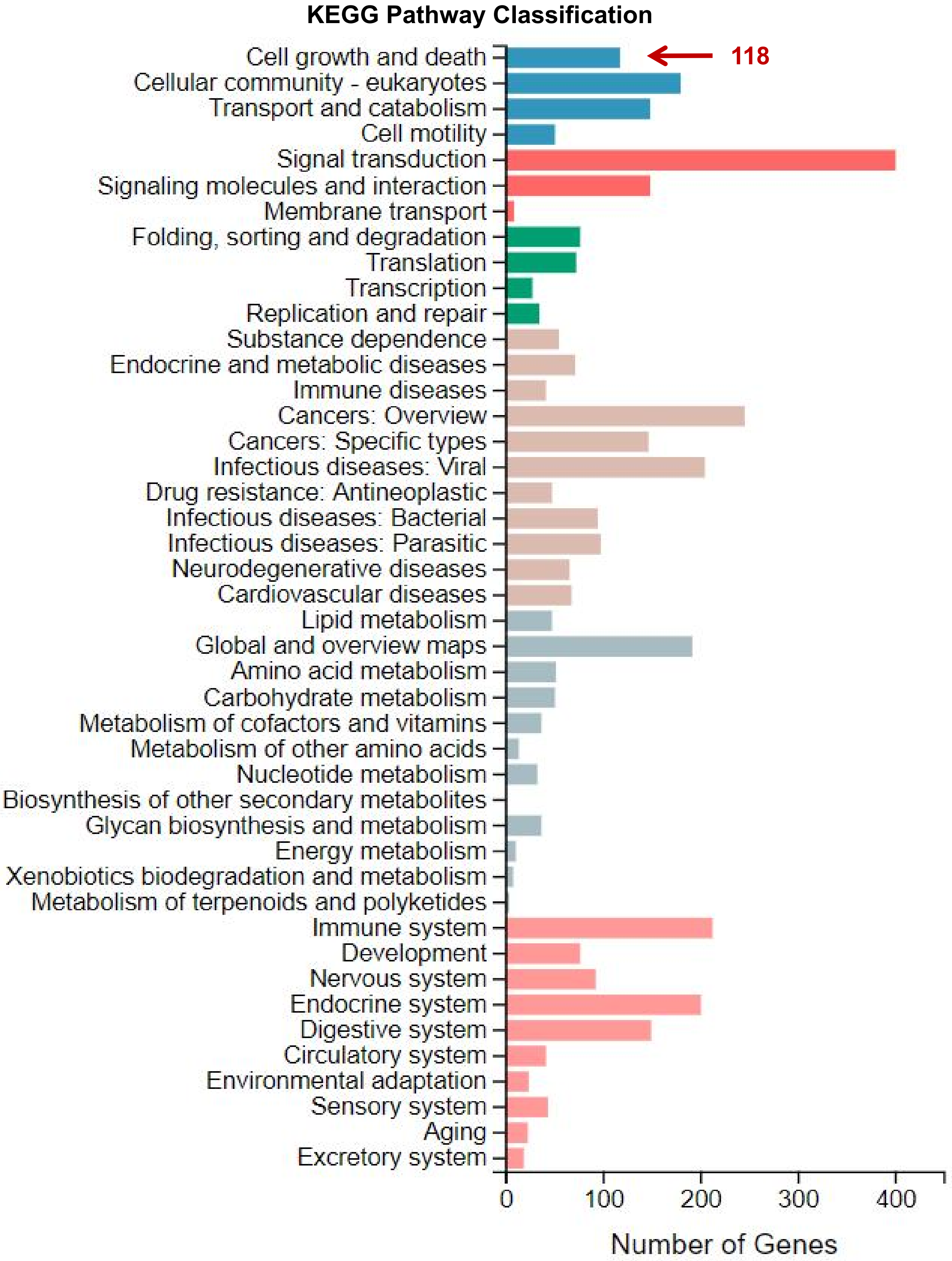
KEGG Pathway Classification
Cell growth and death
Cellular community - eukaryotes
Transport and catabolism
Cell motility
Signal transduction
Signaling molecules and interaction
Membrane transport
Folding, sorting and degradation
Translation
Transcription
Replication and repair
Substance dependence
Endocrine and metabolic diseases
Immune diseases
Cancers: Overview
Cancers: Specific types
Infectious diseases: Viral
Drug resistance: Antineoplastic
Infectious diseases: Bacterial
Infectious diseases: Parasitic
Neurodegenerative diseases
Cardiovascular diseases
Lipid metabolism
Global and overview maps
Amino acid metabolism
Carbohydrate metabolism
Metabolism of cofactors and vitamins
Metabolism of other amino acids
Nucleotide metabolism
Biosynthesis of other secondary metabolites
Glycan biosynthesis and metabolism
Energy metabolism
Xenobiotics biodegradation and metabolism
Metabolism of terpenoids and polyketides
Immune system
Development
Nervous system
Endocrine system
Digestive system
Circulatory system
Environmental adaptation
Sensory system
Aging
Excretory system
118
0
100
200
300
400
Number of Genes

Figure S2

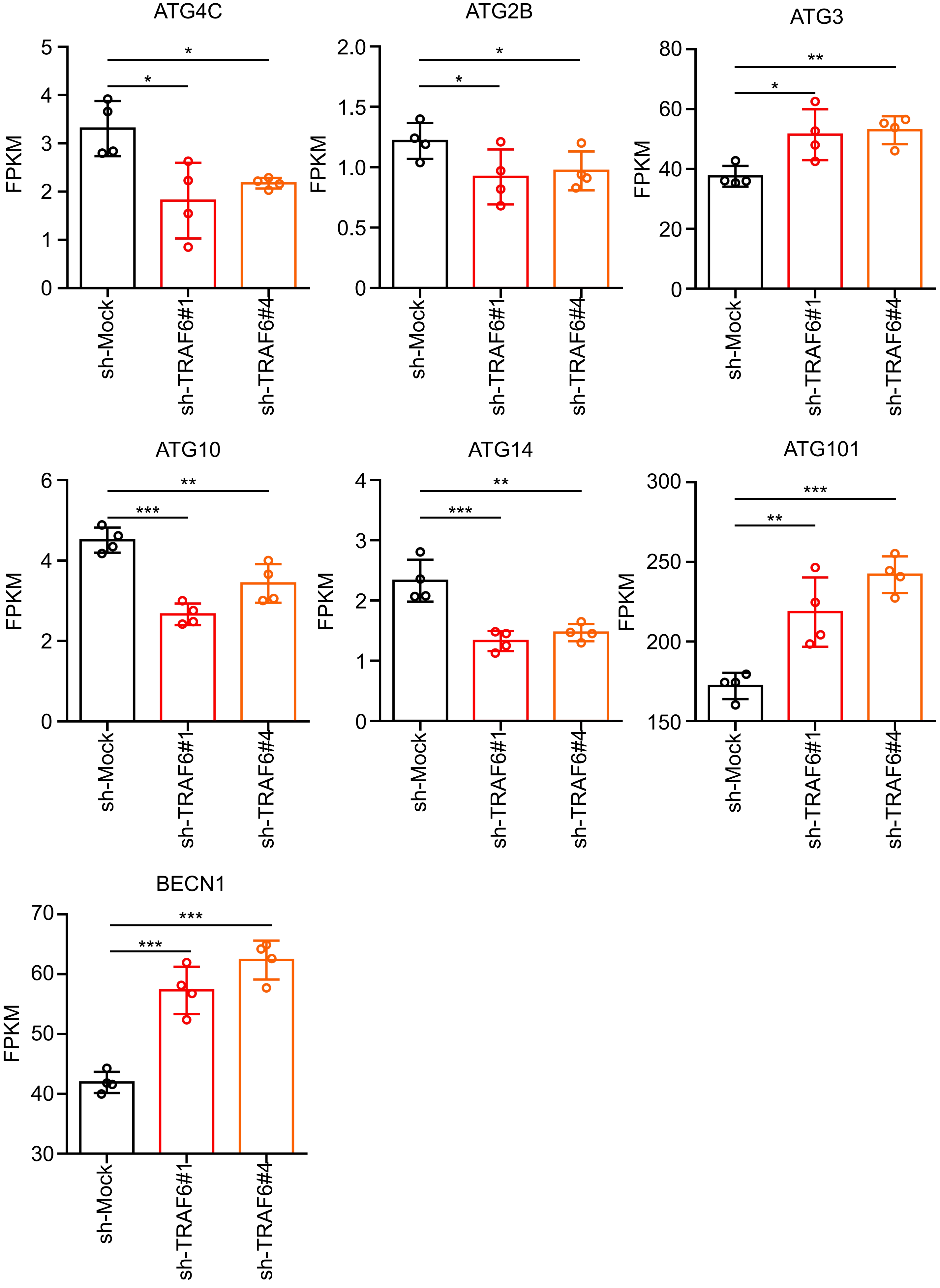
ATG4C
ATG2B
ATG3
ATG10
ATG14
ATG101
BECN1
FPKM
sh-Mock
sh-TRAF6#1
sh-TRAF6#4
*
**
***
0
1
2
3
4
5
0.5
1.0
1.5
2.0
20
40
60
80
6
150
200
250
300
30
50
70

Figure S3

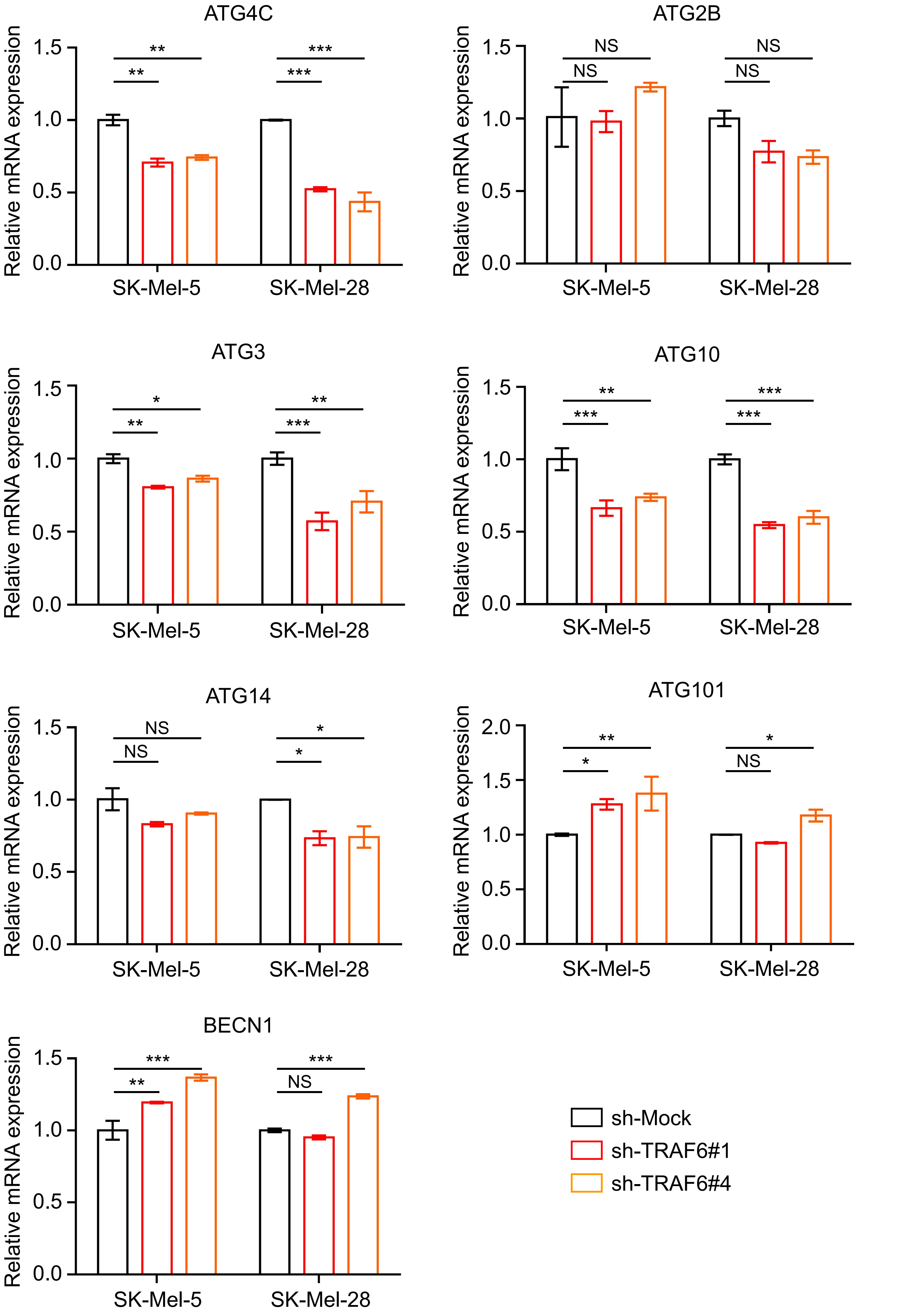

ATG4C
ATG2B
ATG3
ATG10
ATG14
ATG101
BECN1
Relative mRNA expression
SK-Mel-5
SK-Mel-28
0.0
0.5
1.0
1.5
2.0
NS
*
**
***
sh-Mock
sh-TRAF6#1
sh-TRAF6#4

Figure S4

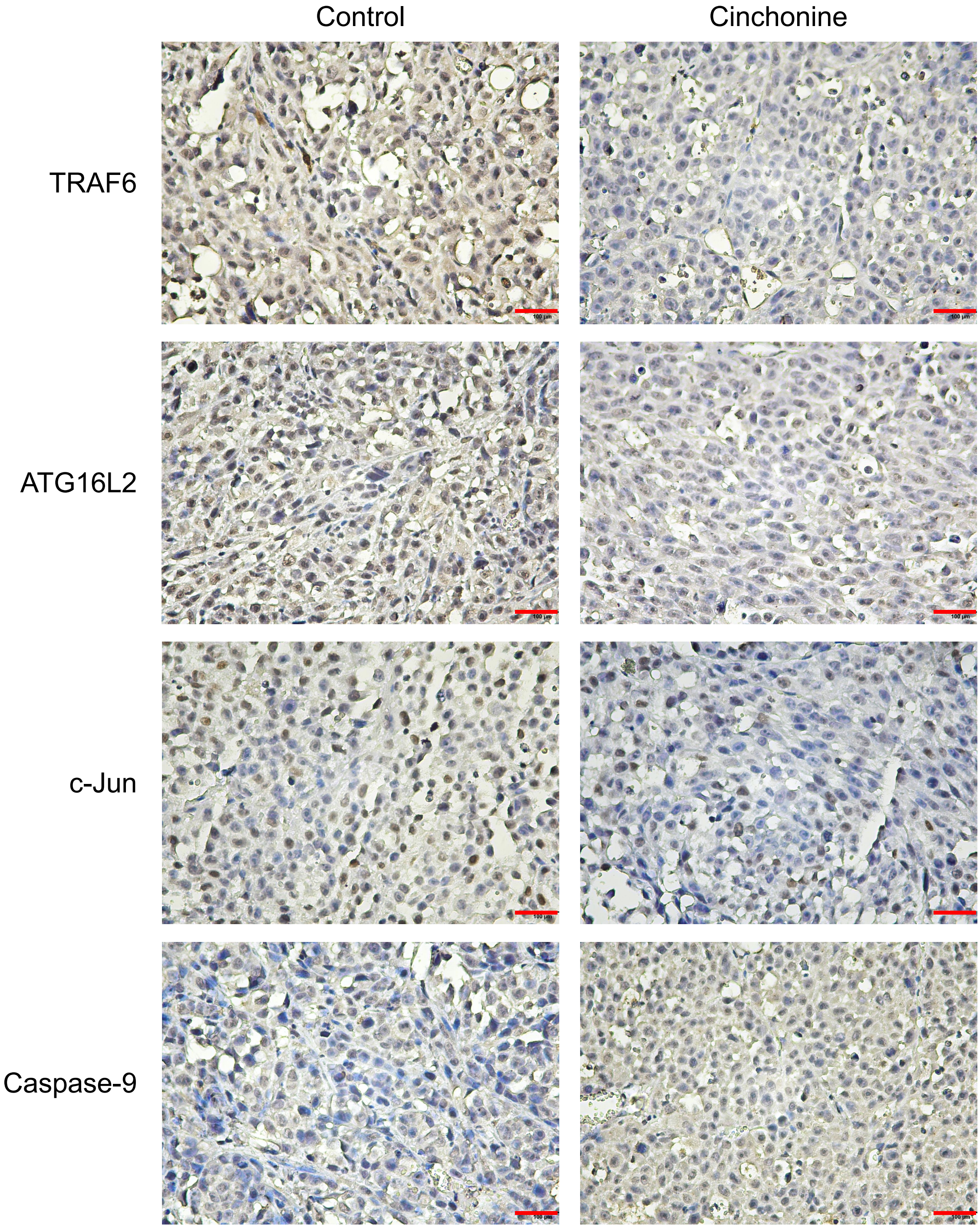

Table S1. Quantitative Real-time PCR primers

Gene	F/R	Sequence	Source
ATG10	Forward	*TGTACTTCATCCCTGCAAGACGAATG*	Sangon
	Reverse	*CTCAGAGGTAGATTCAGCCCAACAAC*	Sangon
ATG101	Forward	*AGCACAGGCAAGTTCCACTACAAG*	Sangon
	Reverse	*TTCCTCAGAAGAGACACGCACATAAG*	Sangon
ATG14	Forward	*GGAGCTGCCGTTATACTGTTCTGG*	Sangon
	Reverse	*GTGTCTCGCCTTTCTCAACCTCTTC*	Sangon
ATG16L2	Forward	*GAGCAGCGATACCAGATCATCC*	Sangon
	Reverse	*CAGCATTGACCTCAGAGAGGTG*	Sangon
ATG2B	Forward	*GCAGTGAAGTCTGATTCTGATGGAGAG*	Sangon
	Reverse	*TCTGATATGGACAACATCGCTGGAAC*	Sangon
ATG3	Forward	*AAGGCATACCTACCAACAGGCAAAC*	Sangon
	Reverse	*CCATCCGCCATCACCATCATCTTC*	Sangon
BECN1	Forward	*GGAGCTGCCGTTATACTGTTCTGG*	Sangon
	Reverse	*GTGTCTCGCCTTTCTCAACCTCTTC*	Sangon
GAPDH	Forward	*GTATCGTGGAAGGACTCATGAC*	Sangon
	Reverse	*ACCACCTTCTTGATGTCATCAT*	Sangon
ChIP Primer 1	Forward	*CAAGTGATCTTTATAATAC*	Sangon
	Reverse	*CAGGTAAGGAGCCATATTG*	Sangon
ChIP Primer 2	Forward	*GTCTGAGAAGCTTGAACTC*	Sangon
	Reverse	*TGCACAGGCCGAGCCTGATG*	Sangon
ChIP Primer 3	Forward	*AGCTCACTGGAAGCAGAG*	Sangon
	Reverse	*AGAGCAGTGCAGGCCCTG*	Sangon
ChIP Primer 4	Forward	*GTTCCCTAGGGATGTTCAAG*	Sangon
	Reverse	*TGCTAGAGAGCCAGCTCTGC*	Sangon
ChIP Primer 5	Forward	*TAGCTAGAGACGGTTTGACC*	Sangon
	Reverse	*TGCAAAGGAGTGGGTAGCTG*	Sangon
